# Case Report: life-threatening haemothorax due to extramedullary haematopoiesis in a patient with Beta-thalassaemia major

**DOI:** 10.3389/fmed.2026.1806378

**Published:** 2026-04-13

**Authors:** Louis Wéry, Romain Niessen, Emilien Ruchonnet, Joao Pinto Pereira

**Affiliations:** 1 Centre Hospitalier Universitaire Vaudois Department of Emergency Medicine, Lausanne, Switzerland; 2 Centre Hospitalier Universitaire Vaudois—Critical Care Department, Lausanne, Switzerland; 3 Centre Hospitalier Universitaire Vaudois Thoracic Surgery Department, Lausanne, Switzerland

**Keywords:** extramedullary haematopoiesis, haemothorax, hemorragic shock, radiotherapy, thalassemia, luspatercept, thoracic surgery

## Abstract

**Introduction:**

Extramedullary haematopoiesis is a rare compensatory phenomenon occurring in haematologic disorders such as thalassaemia. While typically asymptomatic, it can rarely lead to life-threatening complications, including spontaneous haemothorax.

**Case presentation:**

A 60-year-old man with beta-thalassaemia major and a history of extramedullary haematopoiesis presented to the emergency department with acute chest pain, dyspnoea, and hypotension. Imaging revealed a massive left haemothorax secondary to bleeding from a paravertebral extramedullary haematopoiesis mass. The patient was managed with chest tube drainage, vasopressor support, blood transfusions, and low-dose radiotherapy targeted at the bleeding site.

**Discussion:**

Haemothorax is an exceptional complication of extramedullary haematopoiesis. Diagnosis requires a high index of suspicion, and management combines haemodynamic stabilization, surgical or radiological intervention, and long-term control of haematopoiesis. Radiotherapy plays a key role in preventing recurrence.

**Conclusion:**

This case highlights the importance of recognizing extramedullary haematopoiesis as a potential cause of spontaneous haemothorax in patients with thalassaemia and underscores the value of a multidisciplinary approach involving intensive care, hematology, thoracic surgery, and radiation oncology.

## Introduction

Extramedullary haematopoiesis (EMH) is a compensatory mechanism in which blood cell production occurs outside the bone marrow when the bone marrow is no longer able to produce sufficient blood cells (1–3). This compensatory process involves the proliferation of haematopoietic stem cells in reactivated embryonic sites, mainly in the spleen and liver but also in the lymph nodes, posterior mediastinum and thoracic paravertebral tissues (1, 4, 5). It is most commonly associated with chronic haemolytic anaemias such as beta-thalassaemia major, myelofibrosis, and other bone marrow failure syndromes (1, 5, 6). While often asymptomatic, EMH can occasionally lead to mass-effect symptoms, spinal cord compression, or, very rarely, spontaneous hemorrhage (5). Haemothorax due to EMH is an exceptional complication, with only a few cases reported in the literature. The pathophysiology involves the proliferation of erythroid precursors in thoracic sites, forming highly vascularised tumor-like masses that can erode into pleural spaces (2). Here we report a case of life-threatening haemothorax secondary to EMH in a patient with thalassaemia major, discussing its pathophysiology, diagnostic challenges, and multidisciplinary management, thus contributing to the limited body of literature on this severe complication.

## Case description

### Patient information

A 60-year-old man with a known history of beta-thalassaemia major, genetic haemochromatosis, rheumatoid arthritis, and chronic obstructive pulmonary disease.

The patient presented to the emergency department with acute left-sided chest pain, dyspnoea, and dizziness.

### Clinical findings

His vital signs showed hypotension (74/54 mmHg), tachycardia (145 bpm), and hypoxia (SpO_2_ 84% on 2 L/min O_2_). Physical examination revealed decreased breath sounds on the left side and dullness on chest percussion.

### Diagnostic assessment

Initial laboratory tests showed severe anemia (Hb 88 g/L; standard 133–177), thrombocytopenia (113 G/L; standard 150–350), leucocytosis (11.8 g/L; standard 4–10) elevated D-dimers (1,238 ng/ml, standard < 500). Arterial blood gas analysis revealed compensated metabolic acidosis (lactates 4.16 mmol/L; standard 0.5–1.5). Coagulation tests were normal PT: 100% (10.7 s), INR: 1.0, aPTT: 32 s, Fibrinogen: 2.1 g/L, Factor V (110%) and D-dimer (1,238 ng/ml).

A bedside chest X-ray displayed an opacity throughout the left lung field.

Initial differential diagnosis was pneumonia, pleural effusion, alveolar hemorrhage, haemothorax, but no history of trauma or anticoagulation therapy.

**Figure 1:**
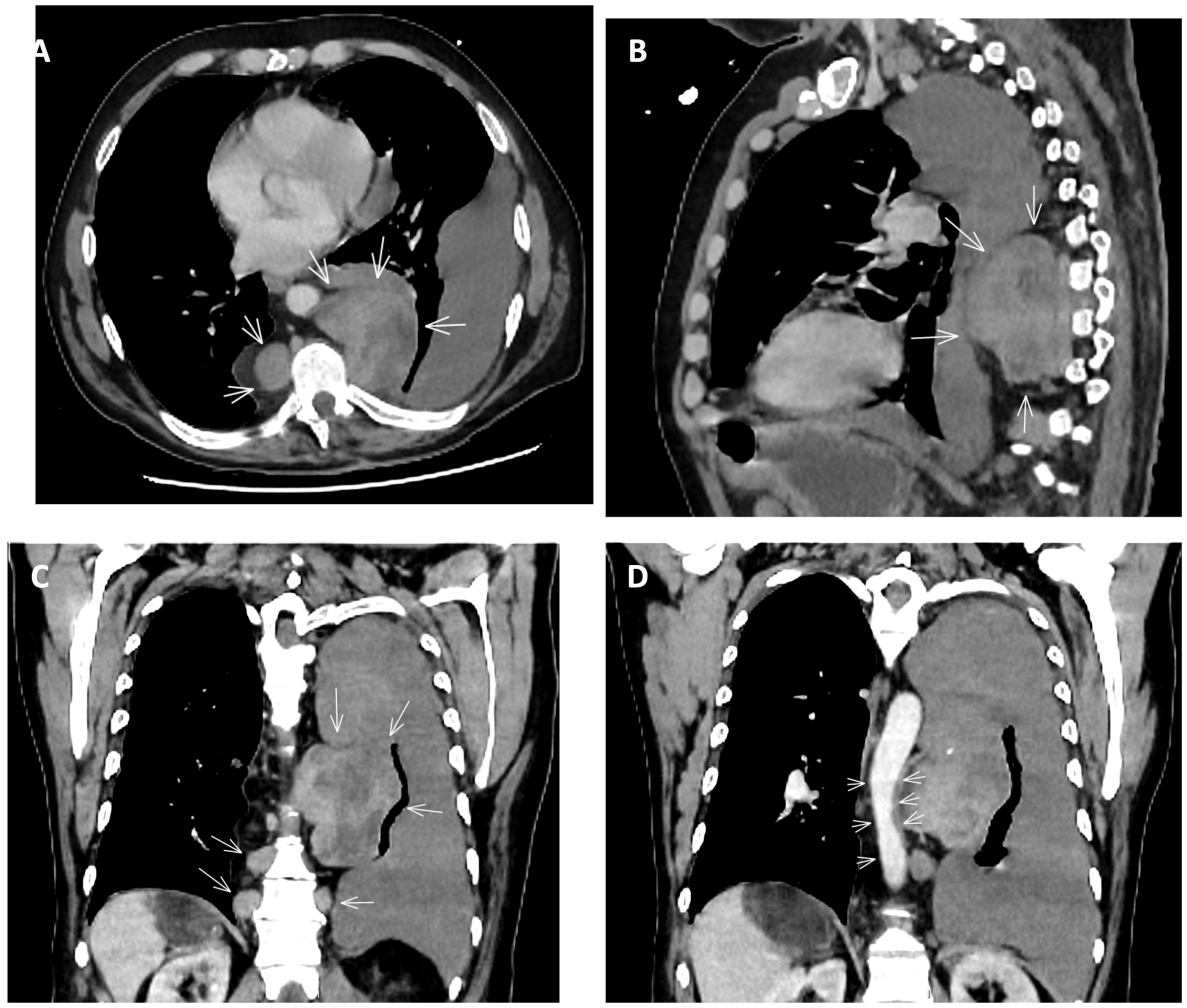
Computed tomographic scan images of the chest revealing **(A–C)** paravertebral soft tissue masses (arrows) consistent with extra-medullary haematopoiesis and an adjacent left-sided haemothorax. **(D)** illustrates aortic compression caused by the large adjacent extramedullary haematopoiesis mass.

**Figure 2:**
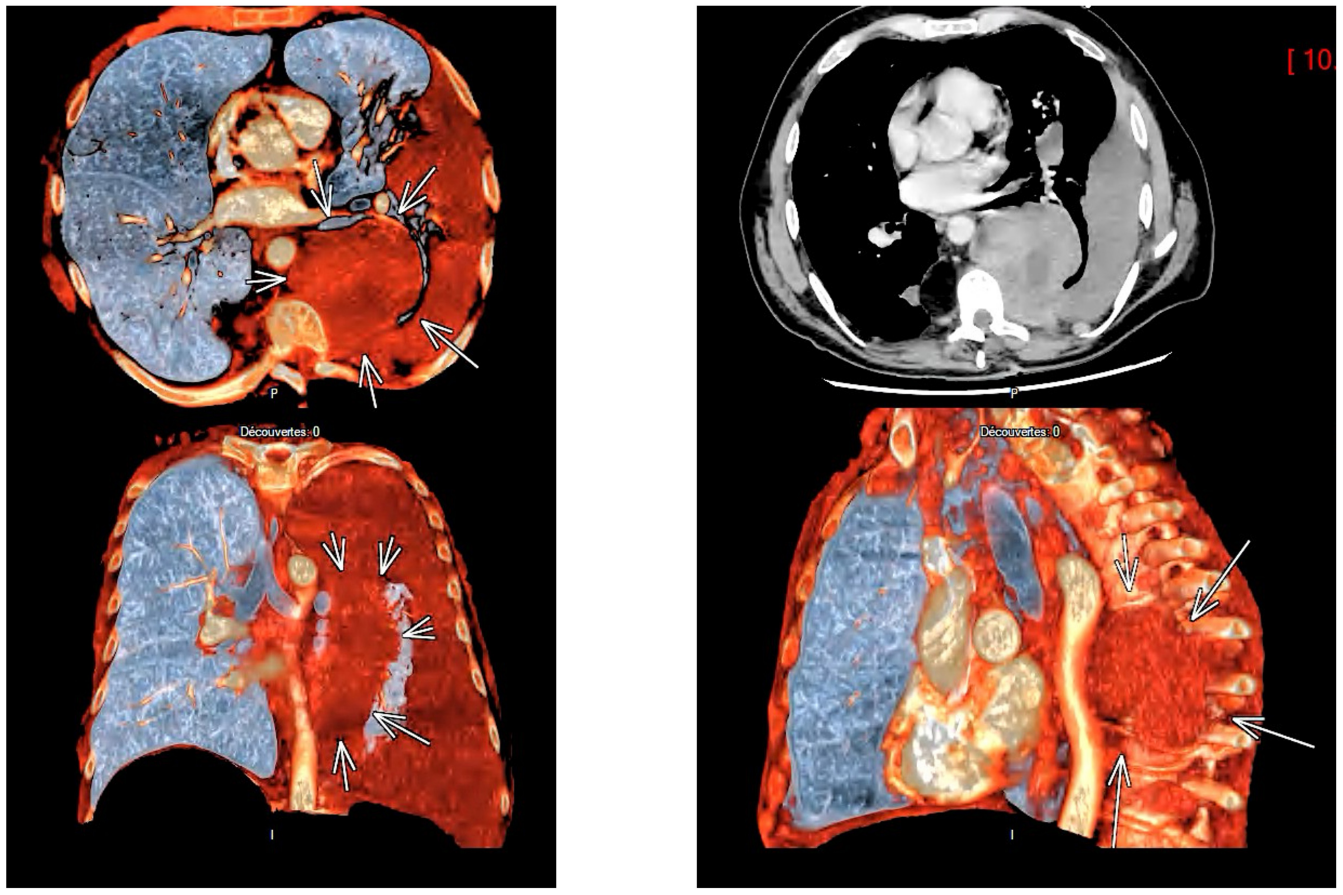
Multiple computed tomographic scan images of the chest with 3D reconstruction, revealing a large paravertebral soft tissue mass (arrows) compatible with extramedullary haematopoiesis and an adjacent left-sided haemothorax.

After stabilization contrast-enhanced CT angiography of the chest and abdomen was performed to characterize this opacity. It demonstrated a large left haemothorax (density of 33 Hounsfield units) with a 9.2 × 7.2 cm hypervascular paravertebral mass, heterogeneous and enhancing in the venous phase, consistent with EMH (Figures 1, 2). Additional findings included hepatosplenomegaly, multiple mediastinal, axillary, and inguinal lymphadenopathies and pleural-based nodules. There was no evidence of pulmonary embolism.

**Figure 3:**
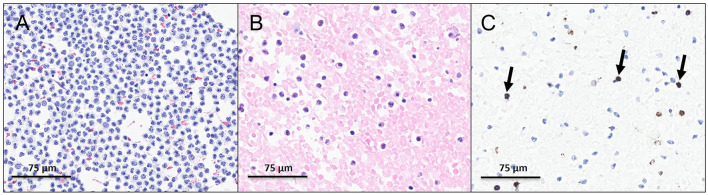
Cytological examination of the pleural effusion. **(A)** Papanicolaou stain (400x) showing numerous neutrophils, lymphocytes, mesothelial cells and erythrocytes. **(B)** Cell block (HE, 400x) demonstrating haemorrhagic background with small basophilic cells. **(C)** CD71 immunostaining (400x) highlighting erythroid precursors (arrows).

Cytological examination of the pleural effusion revealed a highly cellular sample with numerous neutrophils, lymphocytes, mesothelial cells, and erythrocytes (Figure 3A). The cell block showed a haemorrhagic background with the same elements and several small basophilic cells, stained positively for CD71 (Figures 3B, C), consistent with erythroid precursors. No cytological evidence of malignancy was identified.

Biochemical analysis of the pleural fluid revealed the following values: L-lactate 2.44 mmol/L, glucose 6 mmol/L, total protein 32 g/L (with a corresponding serum albumin of 35 g/L), cholesterol 1.0 mmol/L, and lactate dehydrogenase (LDH) 667 U/L (serum LDH: 488 U/L).

These findings are compatible the diagnosis of EMH-related haemothorax.

### Patient management

The patient was initially stabilized with crystalloid infusion.

After CT, noradrenaline was initiated at 4 μg/min and titrated until 10 μg/min. The patient was transfused with two units of red blood cells. A left-sided chest tube was inserted, draining 1.5 L of fresh blood. He was transferred to the intensive care unit for further management where he stayed for 3 days.

In the intensive care unit, hemorrhage management was continued with calcium replacement (calcium gluconate 2 g), fibrinogen replacement (2 g for hypofibrinogenemia at 0.9 g/L; normal range 1.9–4.1) and transfusion of 4 units of red blood cells to raise the hemoglobin level above 100 g/L, in order to limit extramedullary haematopoiesis. The chest tube drained 300 ml of blood within the first 24 h, followed by 250 ml the next day. No antibiotics were administered. Norepinephrine was weaned off within 48 h. On Day 3, he underwent radiotherapy for haemostatic control of the paravertebral mass. As the chest tube output was less than 100 ml that day, it was subsequently removed.

### Follow-up and outcome

The patient was transferred on Day 4 to the thoracic surgery ward, with additional radiotherapy sessions scheduled: in total, the patient received five fractions of 2 Gy each. The patient tolerated the radiotherapy sessions well and the rest of his stay was uneventful, with no recurrence of bleeding (Table 1).

**Table 1:** Overview of key clinical events during hospitalization.

Day	Event
Day 0	ED admission: acute chest pain, dyspnoea, hypotension; vasopressor support (norepinephrine) and crystalloid infusion.
	Chest X-Ray: left opacity.
	CT Angiography: massive haemothorax.
	Norepinephrine initiated and red blood cell transfusion.
	Chest tube insertion (1.5 L of blood).
	ICU admission.
Day 1–2	ICU management and weaning off norepinephrine.
Day 3	Radiotherapy–first session.
Day 4	Transfer to thoracic surgery ward.
Day 7	Radiotherapy–last session. Hospital discharge.

## Discussion

### Extramedullary haematopoiesis and haemothorax

EMH is a well-documented phenomenon in thalassaemia, resulting from chronic erythroid hyperplasia and marrow expansion (2).

The most common sites include the spleen and liver, with hepatosplenomegaly being the most frequent manifestation. Other sites frequently involved include paravertebral regions and lymph nodes, but the pleura may also be involved (1, 3–5).

In the thorax, EMH most often manifests as paravertebral masses which remain asymptomatic in more than 80% of cases (4) but it can also manifest as rib expansion, lung masses, or pulmonary nodules that may cause respiratory failure, dyspnoea, or haemoptysis resulting from alveolar lesions, as well as pleural effusion and/or haemothorax due to pleural involvement (4, 6). EMH is characterized by highly vascularized masses whose histological structure combines haematopoietic tissue and adipose tissue. The abundant vascularization and fragility of these masses make them susceptible to spontaneous rupture (1). Haemothorax is an exceptionally rare complication, usually resulting from rupture of fragile vascularized EMH tissue into the pleural space. Only a handful of cases have been reported, often in patients with thalassaemia intermedia or major (5). The vascular fragility of these masses predisposes to bleeding, particularly in the context of coagulation abnormalities or trauma.

### Diagnostic challenges

In EMH, symptoms are related to the site of involvement (4). EMH-related haemothorax can mimic pulmonary embolism, malignancy, or traumatic injury. The diagnosis is based on chest imaging. The recommended modalities are MRI and CT, with MRI being more sensitive and therefore the modality of choice for diagnosis (4). Imaging allows the identification of paravertebral masses, which in EMH typically appear well-defined and contain fat and haematopoietic tissue (7–11). Active lesions are iso- or hyperintense on T1 and hyperintense on T2 (4, 11). After contrast administration, active masses demonstrate mild homogeneous enhancement due to their high vascularity (4, 12). Histological confirmation is obtained by thoracoscopy or surgical biopsy, as the risk of hemorrhage rupture is too high with percutaneous biopsy (4). Analysis of pleural fluid may reveal hematopoietic elements, notably megakaryocytes, erythroid and myeloid precursors, which can be used to differentiate EMH from simple blood contamination (13, 14). In our case, the combination of known thalassaemia, characteristic paravertebral mass, and haemothorax was strongly suggestive of EMH-related bleeding. No aneurysm, arteriovenous malformation, or cancer-suggestive features were identifed on the CT scan. The cytological confirmation of erythroid precursors in the pleural fluid provided a definitive diagnosis, as previously described in similar cases (1). No malignant cells were seen in the pleural fluid. Microbiological samples remained sterile.

### Management strategies

Management options include blood transfusion, radiotherapy, surgery, hydroxycarbamide (hydroxyurea), or a combination of these modalities. Therapy depends on the patient's clinical status, the severity of symptoms and the size of the mass (5). Asymptomatic lesions may be managed conservatively with close monitoring (1, 4, 5)

First-line treatment involves haemodynamic stabilization and evacuation of blood via chest drainage in case of massive or symptomatic haemothorax. If active bleeding persists or recurs, surgical intervention by video-assisted thoracoscopic surgery (VATS) or thoracotomy may be necessary.

To prevent recurrence, low-dose radiotherapy, with doses typically ranging from 10–30 Gy, targeted at the mass can be effective in reducing mass size and decreasing the risk of hemorrhage by reducing vascularity (2, 4, 5). EMH tissues are highly radiosensitive, and radiotherapy usually produces a rapid therapeutic response.

Blood transfusions are mainstay of treatment, being necessary to maintain higher hemoglobin levels, which suppress compensatory extramedullary haematopoiesis by reducing erythropoietin drive with a target hemoglobin level above 10 g/dL (2, 4).

Cytoreductive agents such as hydroxyurea can reduce haematopoietic drive by stimulating fetal hemoglobin synthesis (2, 4).

Surgery should be reserved for cases that do not respond to transfusions and radiotherapy due to excessive bleeding risk in EMH tissues, which are highly vascularized (4).

Non-hepatosplenic EMH requiring treatment are most commonly and successfully managed with low-dose radiotherapy (5).

The place of Luspatercept, a TGF-β superfamily ligand trap that was recently approved in the USA and Europe to treat anemia in patients with transfusion-dependent β-thalassaemia is not yet clear, and may in some rare situations stimulate EMH and/or splenic sequestration (15).

### Multidisciplinary approach

Effective management of EMH-related haemothorax requires_close collaboration between intensivists, hematologists, radiologists, thoracic surgeons, and radiation oncologists as illustrated by this case.

### Strengths and limitations

This case report presents several strengths. First, it describes an exceptionally rare and life-threatening complication of extramedullary haematopoiesis. The diagnostic process was robust and multimodal, combining a suggestive clinical context, characteristic contrast-enhanced CT findings, and cytological confirmation of erythroid precursors in the pleural fluid, which is infrequently reported and provides strong diagnostic certainty. In addition, the case is thoroughly documented with a clear temporal sequence of events and highlights a successful multidisciplinary management strategy, including haemodynamic stabilization, targeted transfusion therapy and haemostatic radiotherapy, consistent with current pathophysiological understanding and published recommendations. This case illustrates close collaboration between intensivists, hematologists, radiologists, thoracic surgeons, and radiation oncologists. This multidisciplinary approach is key for effective management of EMH-related haemothorax.

However, several limitations must be acknowledged. Long-term follow-up data, including radiological evolution of the extramedullary haematopoietic mass and long-term recurrence risk, are lacking. In addition, alternative therapeutic options such as endovascular embolisation or cytoreductive strategies were not explored in this patient, and the potential long-term impact of radiotherapy could not be assessed.

Despite these limitations, this report provides valuable diagnostic and therapeutic insights and underscores the importance of recognizing extramedullary haematopoiesis as a rare but critical cause of spontaneous haemothorax in patients with chronic haemolytic anemia.

## Conclusion

This case report describes a rare but life-threatening complication of EMH in a patient with thalassaemia major. It underscores the importance of:

- Considering EMH in the differential diagnosis of spontaneous haemothorax in patients with chronic anemia.
- Using CT angiography to rapidly identify the bleeding source.
- Performing cytological examination of pleural fluid for a definitive diagnosis.
- Implementing a multidisciplinary strategy that combines acute resuscitation, transfusion, drainage, and radiotherapy.
- Ensuring long-term monitoring and individualized therapy to prevent recurrence (through red cell transfusion and hydroxyurea).

This case adds to the limited literature on EMH-related haemothorax and highlights the critical role of radiotherapy in its management. Further studies are needed to establish standardized treatment protocols.

## Data Availability

The original contributions presented in the study are included in the article/supplementary material, further inquiries can be directed to the corresponding author.
